# Cue Reactivity in Nicotine and Alcohol Addiction: A Cross-Cultural View

**DOI:** 10.3389/fpsyg.2016.01335

**Published:** 2016-08-31

**Authors:** Wanwan Lv, Qichao Wu, Xiaoming Liu, Ying Chen, Hongwen Song, Lizhuang Yang, Xiaochu Zhang

**Affiliations:** ^1^Chinese Academy of Sciences Key Laboratory of Brain Function and Disease, School of Life Science, University of Science and Technology of ChinaHefei, China; ^2^School of Humanities and Social Science, University of Science and Technology of ChinaHefei, China; ^3^School of Foreign Languages, Anhui Jianzhu UniversityHefei, China; ^4^Center for Biomedical Engineering, School of Information Science and Technology, University of Science and Technology of ChinaHefei, China; ^5^Center of Medical Physics and Technology, Hefei Institutes of Physical Science, Chinese Academy of SciencesHefei, China

**Keywords:** nicotine, alcohol, addiction, cross-culture, cue reactivity, context, emotion, attention

## Abstract

A wealth of research indicates that cue reactivity is critical to understanding the neurobiology of nicotine and alcohol addiction and developing treatments. Functional magnetic resonance imaging (fMRI) and electroencephalograph (EEG) studies have shown abnormal cue reactivity in various conditions between nicotine or alcohol addicts and the healthy. Although the causes of these abnormalities are still unclear, cultural effect can not be ignored. We conduct an review of fMRI and EEG studies about the cue reactivity in nicotine and alcohol addiction and highlight the cultural perspective. We suggest that cultural cue reactivity is a field worth of exploring which may has an effect on addictive behavior through emotion and attention. The cultural role of nicotine and alcohol addiction would provide new insight into understanding the mechanisms of nicotine and alcohol addiction and developing culture-specific therapies. We consider that culture as a context may be a factor that causes confusing outcomes in exploring nicotine and alcohol addiction which makes it possible to control the cultural influences and further contribute to the more consistent results.

## Introduction

Addiction as one of the leading causes of poor health worldwide, has drawn the attention of public including researchers decades ago. Nicotine and alcohol addiction as two of the most common addiction are widely observed because of legalization. Nicotine addiction has been proved to lead to health problems, such as lung cancer, ischemic heart disease and esophageal cancer while alcohol addiction can also cause problems of our health and safety (Castellsagué et al., 1999; Koob and Moal, 2005; Florek and Piekoszewski, 2008; Patel and Feucht, 2010). Additionally, nicotine and alcohol addiction are proved to have an effect on cognition, such as attention and memory (Wiers et al., 2015). Evidence has shown that there existing abnormal functional and structural changes in the brain of nicotine or alcohol addicts (Williams, 1980; Ray et al., 2009; Zhang et al., 2009, 2011a; Vollstädt-Klein et al., 2010a; Bjork and Gilman, 2013).

Although the huge negative influences have been shown, the craving for smoking or drinking in nicotine or alcohol addicts is so difficult to overcome which can lead to relapse over and over again. As a result, the cessation rates remain low and researchers have done a lot to improve a successful cessation with the substance related cue-induced craving which is intense and episodic and can contribute to relapse of addiction (Sinha and Li, 2007; Ferguson and Shiffman, 2009). Although recently, findings show that factor such as trait impulsivity has been considered to partly cause cessation failure (Erblich and Michalowski, 2015) and craving has been proved to be not a necessary condition of relapse (Wray et al., 2013), cue reactivity still plays a very important role in addiction.

Culture as a kind of context may cause differences in behavior (Li et al., 2015). Different cultures may have contributed to different traits in nicotine and alcohol addiction which may provided new sight for the cue reactivity in addiction. We reviewed some reports using EEG and fMRI which try to explain the relationship between culture and the cue-induced activity in nicotine and alcohol addiction.

## Cue Reactivity in Nicotine and Alcohol Addiction

### Cue Reactivity in Smoking or Alcoholic Behavior

As literature has shown, smoking or alcohol-related cues can elicit craving (Erblich and Bovbjerg, 2004; Vollstädt-Klein et al., 2011a; Yarmush et al., 2016) and facilitate the addictive behavior in nicotine or alcohol addicts (Wigmore and Hinson, 1991; Shiffman et al., 2013).

As noted earlier, cue exposure was associated with changes in cognitive function (Bates et al., 2002; Durazzo and Meyerhoff, 2007; Vollstädt-Klein et al., 2009, 2012; Wiers et al., 2013). Studies suggested that addicts show attention bias for the substance-related cues which caused by the expectation of the substance (Field and Cox, 2008; Luijten et al., 2011; Vollstädt-Klein et al., 2011b; Ramirez et al., 2014) and addicts represent the substance-related pathological memory with which the substance-related cues might obtain the power to motivate (Hyman, 2005).

In all, exposed to smoking or alcohol-related cues, smokers or alcoholics change their behavioral patterns. It seems that nicotine and alcohol addicts are susceptible to the substance-related context which may plays an important part in the abnormal addictive process and is worthy of exploring.

### Cue Reactivity in Neural Responses in Nicotine and Alcohol Addiction

#### Cue Reactivity with EEG

As non-invasive measure of human brain function, EEG technique has been widely used in nicotine and alcohol addiction to detect specific sensory and cognitive event to specific cues. What’s more, the P3 of the event-related potentials (ERPs) related to the involvement of motivational and arousal systems (Petit et al., 2015) and the Slow Positive Waves (SPWs) of the ERPs which reflect the brain’s activation of motivational systems to emotional cues (Cuthbert et al., 2000) were most suggested. It is indicated that both P3 and SPW amplitudes in response to smoking cues are significantly more enhanced in smokers than in the healthy at frontal and central sites indicating that smokers show more attention bias for smoking cues (Namkoong et al., 2004; Littel and Franken, 2007; Marianne et al., 2012).

The larger amplitude of P3 was also observed in non-smokers in response to smoking cues than neutral cues (Mcdonough and Warren, 2001) while non-alcoholics show no significant differences in P3 amplitudes even though the larger P3 was observed in alcoholics when faced with alcohol-related cues than neural cues (Namkoong et al., 2004). One explanation is that non-smokers may get hurt from the secondhand smoking so that they are also sensitive to smoking cues while the non-alcoholics are not always concerned about alcohol given that the alcohol cannot hurt them directly. It seems that addiction has lead to a bit different consequences in motivation and arousal which may be caused by the different context between nicotine and alcohol addiction.

#### Cue Reactivity with fMRI

Abundant fMRI evidences have suggested that cue-induced reactivity among nicotine or alcohol addicts is different from the healthy which shows functional and structural abnormalities in the brain of addicts as stated earlier. Furthermore, brain regions associated with anterior cingulate cortex (ACC) and other regions in attentional network including orbitofrontal prefrontal cortex, default mode networks involved precuneus, salience network including insula and the reward network refered to amygdala and striatum appear to be stimulated abnormally by smoking cues (Due et al., 2002; Brody et al., 2007; Franklin et al., 2011; Zhang et al., 2011b) and alcohol-related cues (Vollstädt-Klein et al., 2010b; Claus et al., 2013; Dager et al., 2014).

With these results, researchers have explored the important functions of these regions in nicotine and alcohol addiction (Vollstädt-Klein et al., 2010b; Zhang et al., 2011a; Hayashi et al., 2013; Lerman et al., 2014). However, whether the role these regions played in nicotine and alcohol addiction are same or not is an open question.

The addiction severity of nicotine has been reported negatively correlated with smoking cue-induced activity in amygdala (Vollstädt-Klein et al., 2011a) while evidence showed that the severity of alcohol was positively associated with amygdala response to alcohol-related cues among alcoholics (Claus et al., 2011). As researches on amygdala showed significant association with the processing of learned reward values of conditioned cues and context (Robbins et al., 2008), the different role of amygdala in nicotine and alcohol addiction indicated that the sensitivity to context between smokers and alcoholics is different. As a result, context plays an important role in the formation of nicotine and alcohol addiction.

In addition, alcohol-related cue-induced activity in the ventral striatum showed positive correlation with self-reported alcohol craving (Myrick et al., 2004; Seo et al., 2011). As the correlation between cigarette craving and ventral striatum response to smoking cues yielded mixed results, with both negative correlations (Mcclernon et al., 2005) and null correlations (David et al., 2005) which is different from the results in alcohol addiction, we can infer that the ventral striatum plays different roles in the craving for smoking or drinking. The ventral striatum has been reported to make great contribution to behavioral conditioning reflecting the reward value predicted by discriminative cues (Schultz et al., 1997). It seems that the reward value of smoking cues to smokers is different from that of alcohol-related cues to alcoholics which may be due to the different craving elicited by the different context around them.

## Cultural Effect In General

### General Cultural Effect on Behaviors

Behavioral differences between Eastern and Western cultures has been explored widely. It’s said that East Asians and Asian-Americans pay more attention to the background than European Americans who tend to focus on the foreground and focal objects with several tasks (Goto et al., 2013). In line with that, East Asians are likely to attribute the behavior to social context while Westerners tend to attribute the same thing to their internal dispositions (Choi et al., 1998). Nisbett analyzed these findings and concluded that East Asian’ cognitive styles are characterized as more holistic and European American cognitive styles are characterized as more analytic (Nisbett et al., 2001; Nisbett and Miyamoto, 2005). Besides, as for the social behavior, East Asians were interdependent and collectivism whereas Westerns were independent and individualism (Suh et al., 1998). Culture is closely related to our behavioral patterns and different cultures can induce different behavioral patterns.

### Cultural Effect on Neural Responses

#### Cultural Effect with EEG

Research on cognition beyond observable behavioral performance with EEG revealed some neural differences in different cultures. To our knowledge, most of the research results are related to the Eastern and Western cultures indicating the huge differences between the two distinctive cultures while the cultural differences among different Western cultures are seldom revealed. Research results suggested that there is cultural effect in motivational responses to felt misunderstanding (Lun et al., 2010). Besides, there are fundamental differences in the distribution of EEG between Easterners and Westerners as EEG asymmetry between Japanese and Westerners is different (Moss et al., 1985).

One study utilizing the good time solution of EEG suggested that Chinese and Italian attend the commercials with the same theme in different moment reflecting the different span of attention (Vecchiato et al., 2011). Although the early ERP components in the processing of emotional pictures is not modulated by the cultures in European and Japanese, the late stage of the processing is different (Fleming et al., 2010). Further, huge evidence about cognitive differences between East and West with respect to emotion such as semantic affective processing (Goto et al., 2013) and expression of emotion with the N400 (Liu et al., 2015), and emotion suppression by the parietal late positive potential (Murata et al., 2012) indicating that Easterners are more sensitive to the relationship between context and objects than Westerners. In addition to emotion, the most discussed was cultural differences in self-construal which may be different from the processing of the emotion by the differences in N400, P3 and the early component N170 in various conditions (Lewis et al., 2008; Vizioli et al., 2010; Masuda et al., 2014).

#### Cultural Effect with fMRI

Quantitative fMRI studies has been conducted to examine cultural differences. Results have revealed specific regions related to cultural effect between Eastern and Western cultures in the neural responses to various stimulus. However, given the variation of the culture-specific task, the cultural effect can be concluded in general that social cognitive tasks such as cultural self-referential and self-representation are related the responses in the medial prefrontal cortex and ACC (Zhu et al., 2007; Sul et al., 2012). Non-social cognitive tasks such as processing of faces and attentional control are associated with the activation of attentional network (Gutchess et al., 2006; Hedden et al., 2008; Goh et al., 2010). Social effective tasks such as emotional pain perception, recognition of emotions, empathy, and fearful faces may refer to the different activation patterns of insula, amygdala and ACC (Moriguchi et al., 2005; Chiao et al., 2008; Derntl et al., 2009, 2012; Cheon et al., 2011, 2013; Greck et al., 2012).

Studies focus on the emotion among different Western cultures also have disclosed some rules. Firstly, there are similar neural activity in amygdala which is associated with emotion when viewing image of same or other-race faces among African-American and Caucasian-American individuals (Lieberman et al., 2005). However, Western cultures also showed neural differences which may be related to emotion as the fact that the neural correlation of empathic resonance for pain in participants with different Western cultures is different (Azevedo et al., 2013). Besides, during a social evaluative task, it is concluded that White Americans generally exhibited more activation in regions associated with conflict resolution and cognitive control, while African Americans (AA) exhibited activation mostly in areas associated with emotion and memory (Greer et al., 2012).

#### Summary

In all, culture between Eastern and Western have shown differences with respect to attention, emotion and self-construal while studies on Western cultural differences focus on the emotion factors. It seems that the emotional and attentional factors play important roles in general cultural differences while the self-construal-related context may play an essential role in cultural difference between Eastern and Western cultures but not among different Western cultures.

## Cultural Effect in Nicotine and Alcohol Addiction

### Cultural Effect on Addictive Behaviors in Nicotine and Alcohol Addiction

Given the large influences on behavior stated earlier, culture may have an effect on addictive behaviors. Some direct evidences have shown that culture is a factor influencing the use behavior of alcohol (Zane and Sasao, 2010; Krentzman et al., 2012) and cigarette (Benowitz, 1996; Florek and Piekoszewski, 2008; Coleman-Cowger and Catlin, 2013). For example, AA smokers smoke fewer cigarettes per day, metabolize nicotine much lower, prefer mentholated cigarettes and have a higher level of nicotine dependence than Caucasians (CC) smokers (Benowitz, 1996; Coleman-Cowger and Catlin, 2013). As a result, it is more difficult for AA smokers to make a successful quit than CC smokers, although they were reported to make more quit attempts (Fiore et al., 1989). In addition, different cultures have caused various views on smoking (Finkenauer et al., 2009) or drinking (Caetano et al., 1998) and different motivation for smoking (Sánchez-Johnsen et al., 2006) or drinking (Nagoshi et al., 1994; Piko, 2007).

Take the essential emotion factors in nicotine and alcohol addiction and the fact that perceptions of emotion are not culturally universal (Gendron et al., 2014) into consideration, culture may be a factor that influences the neural responses of different addiction. For example, smoking and drinking in some Eastern cultures like Chinese are just a form of social contact which involves complex emotion while in Western cultures things are different as people usually smoke or drink by themselves. Given the fact stated above, the emotion factors in different cultures of nicotine and alcohol addiction may not always be the same.

Additionally, there are actually disparities of cognition for addicts regrading to different cultures. To our knowledge, a research showed that Dutch adolescents had an avoidance bias toward both smoking and neutral pictures, while American adolescents didn’t have a significant bias toward either smoking or neutral pictures (Larsen et al., 2014).

Above all, culture has an effect on addictive behavior in nicotine and alcohol addiction and both emotion and attention which are related to cue reactivity play important roles in the cultural effect. It seems that cultural effect on cue reactivity of smokers and alcoholics are associated with emotion and attention.

### Cultural Effect on Neural Responses in Nicotine and Alcohol Addiction

Furthermore, studies focusing on different neural responses among cross-cultural smokers have shown that cultural difference is a possible factor in modifying the effect of nicotine. Okuyemi et al. (2006) has done a research to examine whether smokers from different cultural background process smoking-related cues in a different way using fMRI. The experiment were conducted among AA and CC smokers which is related to racial effect. However, as the ethnicity is not the cause but the result of cultural phenomenon (Claude, 1952), it also have proved a strong cultural effect on neural responses by showing that the brain activation to smoking cues (versus neutral cues) of AA smokers is stronger than CC smokers in several priori regions of interest.

In addition, the disparities of cultural neural responses can also be tracked by the comparison of some studies. A study suggested that chronic smokers from China showed decreased activation in the left thalamus (Liao et al., 2012), while another study suggested smokers from Australia showed decreased thalamus activation in the right side (Almeida et al., 2008). It seems that the activation of several regions are different among smokers or alcoholics with different cultures which should be further studied.

### Summary

Overall, culture as a kind of context influences the behavior and the neural activity in addiction and there may be two main ways. Firstly, culture affects the emotion of individuals which may causes different arousing to the emotional factors of the cues and elicits the culture-specific neural responses in nicotine and alcohol addiction. Secondly, culture may has an effect on the attention of the nicotine and alcohol addicts. And with the two factors associated with cue reactivity in nicotine and alcohol addiction, culture may have an effect on the cue reactivity.

## Unsolved Problems

Although, we have found that culture could influence the cue reactivity in nicotine and alcohol addiction which is related to emotion and attention, some questions are still unclear. Firstly, causes of the differences between nicotine and alcohol addiction are uncertain. Although, we have supposed that the different context may be the reason, we can not exclude the possibility that nicotine and alcohol have different effects on nervous system given that there is no research comparing the effects of these incompatible substances directly. Secondly, we found that the differences between Western and Eastern cultures are more significant than among Western cultures, which we speculated may be caused by more commonalities within Western cultures. But it still needs more targeted research. Thirdly, there are cultural effect on nicotine and alcohol addiction, but literature focusing on the cultural effects on neural responses in nicotine and alcohol addiction is rare, the neural basis is far from fully understood. In addition, we have known that addicts with different cultures have different responses to the substance-related cues, but the cues used in these researches are not the same which may relate to culture-specific factors. Whether the addicts have stronger responses to the culture-specific cues still need further research.

With the solution to these questions, we could have a better understanding of the cultural effect on nicotine and alcohol addiction and the neural mechanisms of them, which would be beneficial to improve the treatments by developing culture-specific treatments of addiction. Besides, understanding the cultural effect would make it possible to control the cultural factor to resolve the inconsistencies caused by culture. At last, objective differences in neural responses caused by cultural effect may improve our rational knowledge of cultural differences in general which may further contribute to the elimination of misunderstandings among different cultures.

## Author Contributions

XZ supervised this study and revised each draft. WL and QW wrote this paper. XL, YC, HS, and LY provided suggestions on this paper.

## Conflict of Interest Statement

The authors declare that the research was conducted in the absence of any commercial or financial relationships that could be construed as a potential conflict of interest.

## References

[B1] Almeida O. P. Garrido G. J. Lautenschlager N. T. Hulse G. K. Jamrozik K. Flicker L. (2008). Smoking is associated with reduced cortical regional gray matter density in brain regions associated with incipient Alzheimer disease. *Am. J. Geriatr. Psychiatry* 16 92–98. 10.1097/JGP.0b013e318157cad2 18165464

[B2] Azevedo R. T. Macaluso E. Avenanti A. Santangelo V. Cazzato V. Aglioti S. M. (2013). Their pain is not our pain: brain and autonomic correlates of empathic resonance with the pain of same and different race individuals. *Hum. Brain Mapp.* 34 3168–3181. 10.1002/hbm.22133 22807311 PMC6870096

[B3] Bates M. E. Bowden S. C. Barry D. (2002). Neurocognitive impairment associated with alcohol use disorders: implications for treatment. *Exp. Clin. Psychopharmacol.* 10 193–212. 10.1037/1064-1297.10.3.193 12233981

[B4] Benowitz N. L. (1996). Cotinine as a biomarker of environmental tobacco smoke exposure. *Epidemiol. Rev.* 18 188–204. 10.1093/oxfordjournals.epirev.a017925 9021312

[B5] Bjork J. M. Gilman J. M. (2013). The effects of acute alcohol administration on the human brain: insights from neuroimaging. *Neuropharmacology* 84 101–110. 10.1016/j.neuropharm.2013.07.039 23978384 PMC3971012

[B6] Brody A. L. Mandelkern M. A. Olmstead R. E. Jou J. Tiongson E. Allen V. (2007). Neural substrates of resisting craving during cigarette cue exposure. *Biol. Psychiatry* 62 642–651. 10.1016/j.biopsych.2006.10.026 17217932 PMC1992815

[B7] Caetano R. Clark C. L. Tam T. (1998). Alcohol consumption among racial/ethnic minorities: theory and research. *Alcohol Health Res. World* 22 233–241. 15706749 PMC6761890

[B8] Castellsagué X. Muñoz N. De Stefani E. Victora C. G. Castelletto R. Rolón P. A. (1999). Independent and joint effects of tobacco smoking and alcohol drinking on the risk of esophageal cancer in men and women. *Int. J. Cancer* 82 657–664. 10.1002/(SICI)1097-0215(19990827)82:5<657::AID-IJC7>3.0.CO;2-C 10417762

[B9] Cheon B. K. Im D. M. Harada T. Kim J. S. Mathur V. A. Scimeca J. M. (2011). Cultural influences on neural basis of intergroup empathy. *Neuroimage* 57 642–650. 10.1016/j.neuroimage.2011.04.031 21549201

[B10] Cheon B. K. Im D. M. Harada T. Kim J. S. Mathur V. A. Scimeca J. M. (2013). Cultural modulation of the neural correlates of emotional pain perception: the role of other-focusedness. *Neuropsychologia* 51 1177–1186. 10.1016/j.neuropsychologia.2013.03.018 23566889

[B11] Chiao J. Y. Gordon I. H. L. Nogawa J. Bar M. Aminoff E. Sadato N. (2008). Cultural specificity in amygdala response to fear faces. *J. Cogn. Neurosci.* 20 2167–2174. 10.1162/jocn.2008.20151 18457504

[B12] Choi I. Nisbett R. E. Norenzayan A. (1998). Causal attribution across cultures: variation and universality. *Psychol. Bull.* 125 47–63. 10.1037/0033-2909.125.1.47

[B13] Claude L. S. (1952). *Race et Histoire. Le Racisme Devant La Science.* Paris: UNESCO.

[B14] Claus E. D. Blaine S. K. Filbey F. M. Mayer A. R. Hutchison K. E. (2013). Association between nicotine dependence severity, BOLD response to smoking cues, and functional connectivity. *Neuropsychopharmacology* 38 2363–2372. 10.1038/npp.2013.134 23708507 PMC3799055

[B15] Claus E. D. Ewing S. W. Filbey F. M. Sabbineni A. Hutchison K. E. (2011). Identifying neurobiological phenotypes associated with alcohol use disorder severity. *Neuropsychopharmacology* 36 2086–2096. 10.1038/npp.2011.99 21677649 PMC3158325

[B16] Coleman-Cowger V. H. Catlin M. L. (2013). Changes in tobacco use patterns among adolescents in substance abuse treatment. *J. Subst. Abuse Treat.* 45 227–234. 10.1016/j.jsat.2013.02.004 23537924

[B17] Cuthbert B. N. Schupp H. T. Bradley M. M. Birbaumer N. Lang P. J. (2000). Brain potentials in affective picture processing: covariation with autonomic arousal and affective report. *Biol. Psychol.* 52 95–111. 10.1016/S0301-0511(99)00044-7 10699350

[B18] Dager A. D. Anderson B. M. Rosen R. Khadka S. Sawyer B. Jiantonio-Kelly R. E. (2014). Functional magnetic resonance imaging (fMRI) response to alcohol pictures predicts subsequent transition to heavy drinking in college students. *Addiction* 109 585–595. 10.1111/add.12437 24304235 PMC3951577

[B19] David S. P. Munafò M. R. Johansen-Berg H. Smith S. M. Rogers R. D. Matthews P. M. (2005). Ventral striatum/nucleus accumbens activation to smoking-related pictorial cues in smokers and nonsmokers: a functional magnetic resonance imaging study. *Biol. Psychiatry* 58 488–494. 10.1016/j.biopsych.2005.04.028 16023086 PMC4439461

[B20] Derntl B. Habel U. Robinson S. Windischberger C. Kryspin-Exner I. Gur R. C. (2009). Amygdala activation during recognition of emotions in a foreign ethnic group is associated with duration of stay. *Soc. Neurosci.* 4 294–307. 10.1080/17470910802571633 19479638

[B21] Derntl B. Habel U. Robinson S. Windischberger C. Kryspin-Exner I. Gur R. C. (2012). Culture but not gender modulates amygdala activation during explicit emotion recognition. *BMC Neurosci.* 13:54 10.1186/1471-2202-13-54 PMC3404024 22642400

[B22] Due D. L. Huettel S. A. Hall W. G. Rubin D. C. (2002). Activation in mesolimbic and visuospatial neural circuits elicited by smoking cues: evidence from functional magnetic resonance imaging. *Am. J. Psychiatry* 159 954–960. 10.1176/appi.ajp.159.6.954 12042183

[B23] Durazzo T. C. Meyerhoff D. J. (2007). Neurobiological and neurocognitive effects of chronic cigarette smoking and alcoholism. *Front. Biosci.* 12:4079-100. 10.2741/2373 17485360

[B24] Erblich J. Bovbjerg D. H. (2004). In vivo versus imaginal smoking cue exposures: is seeing believing? *Exp. Clin. Psychopharmacol.* 12 208–215. 10.1037/1064-1297.12.3.208 15301638

[B25] Erblich J. Michalowski A. (2015). Impulsivity moderates the relationship between previous quit failure and cue-induced craving. *Addict. Behav.* 51 7–11. 10.1016/j.addbeh.2015.06.044 26183443 PMC4558227

[B26] Ferguson S. G. Shiffman S. (2009). The relevance and treatment of cue-induced cravings in tobacco dependence. *J. Subst. Abuse Treat.* 36 235–243. 10.1016/j.jsat.2008.06.005 18715743

[B27] Field M. Cox W. M. (2008). Attentional bias in addictive behaviors: a review of its development, causes, and consequences. *Drug Alcohol Depend* 97 1–20. 10.1016/j.drugalcdep.2008.03.030 18479844

[B28] Finkenauer R. Pomerleau C. S. Snedecor S. M. Pomerleau O. F. (2009). Race differences in factors relating to smoking initiation. *Addict. Behav.* 34 1056–1059. 10.1016/j.addbeh.2009.06.006 19595515 PMC2753519

[B29] Fiore M. C. Novotny T. E. Pierce J. P. Hatziandreu E. J. Patel K. M. Davis R. M. (1989). Trends in cigarette smoking in the United States. The changing influence of gender and race. *JAMA* 261 49–55. 10.1001/jama.1989.03420010059033 2908994

[B30] Fleming K. K. Bandy C. L. Kimble M. O. (2010). Decisions to shoot in a weapon identification task: the influence of cultural stereotypes and perceived threat on false positive errors. *Soc. Neurosci.* 5 201–220. 10.1080/17470910903268931 19813139 PMC4214075

[B31] Florek E. Piekoszewski W. (2008). [Pharmacotherapy of smoking cessation with application of nicotine and nicotine free drugs]. *Prz. Lek.* 65 700–705. 19189582

[B32] Franklin T. Wang Z. Suh J. J. Hazan R. Cruz J. Li Y. (2011). Effects of varenicline on smoking cue-triggered neural and craving responses. *Arch. Gen. Psychiatry* 68 516–526. 10.1001/archgenpsychiatry.2010.190 21199958 PMC3743240

[B33] Gendron M. Roberson D. van der Vyver J. M. Barrett L. F. (2014). Perceptions of emotion from facial expressions are not culturally universal: evidence from a remote culture. *Emotion* 14 251–262. 10.1037/a0036052 24708506 PMC4752367

[B34] Goh J. O. Leshikar E. D. Sutton B. P. Tan J. C. Sim S. K. Hebrank A. C. (2010). Culture differences in neural processing of faces and houses in the ventral visual cortex. *Soc. Cogn. Affect. Neurosci.* 5 227–235. 10.1093/scan/nsq060 20558408 PMC2894673

[B35] Goto S. G. Yee A. Lowenberg K. Lewis R. S. (2013). Cultural differences in sensitivity to social context: detecting affective incongruity using the N400. *Soc. Neurosci.* 8 63–74. 10.1080/17470919.2012.739202 23116082

[B36] Greck M. D. Shi Z. Wang G. Zuo X. Yang X. Wang X. (2012). Culture modulates brain activity during empathy with anger. *Neuroimage* 59 2871–2882. 10.1016/j.neuroimage.2011.09.052 21983057

[B37] Greer T. M. Vendemia J. M. C. Stancil M. (2012). Neural correlates of race-related social evaluations for African Americans and White Americans. *Neuropsychology* 26 704–712. 10.1037/a0030035 23106117

[B38] Gutchess A. H. Welsh R. C. Boduroglu A. Park D. C. (2006). Cultural differences in neural function associated with object processing. *Cogn. Affect. Behav. Neurosci.* 6 102–109. 10.3758/CABN.6.2.102 17007231

[B39] Hayashi T. Ji H. K. Strafella A. P. Dagher A. (2013). Dorsolateral prefrontal and orbitofrontal cortex interactions during self-control of cigarette craving. *Proc. Natl. Acad. Sci. U.S.A.* 110 4422–4427. 10.1073/pnas.1212185110 23359677 PMC3600476

[B40] Hedden T. Ketay S. Aron A. Markus H. R. Gabrieli J. D. (2008). Cultural influences on neural substrates of attentional control. *Psychol. Sci.* 19 12–17. 10.1111/j.1467-9280.2008.02038.x 18181784

[B41] Hyman S. E. (2005). Addiction: a disease of learning and memory. *Am. J. Psychiatry* 162 1414–1422. 10.1176/appi.ajp.162.8.1414 16055762

[B42] Koob G. F. Moal M. L. (2005). *Neurobiology of Addiction.* Cambridge, MA: Elsevier Academic.

[B43] Krentzman A. R. Battle D. Pagano M. E. Andrade F. H. Bradley J. C. Delva J. (2012). The role of religiousness on substance-use disorder treatment outcomes: a comparison of Black and White Adolescents. *J. Soc. Social Work Res.* 3 113–128. 10.5243/jsswr.2012.8 22970338 PMC3437261

[B44] Larsen H. Kong G. Becker D. Cousijn J. Boendermaker W. Cavallo D. (2014). Implicit motivational processes underlying smoking in american and dutch adolescents. *Front. Psychiatry* 5:51 10.3389/fpsyt.2014.00051 PMC4032927 24904435

[B45] Lerman C. Gu H. Loughead J. Ruparel K. Yang Y. Stein E. A. (2014). Large-scale brain network coupling predicts acute nicotine abstinence effects on craving and cognitive function. *JAMA Psychiatry* 71 523–530. 10.1001/jamapsychiatry.2013.4091 24622915 PMC4097018

[B46] Lewis R. S. Goto S. G. Kong L. L. (2008). Culture and context: East Asian American and European American differences in P3 event-related potentials and self-construal. *Pers. Soc. Psychol. Bull.* 34 623–634. 10.1177/0146167207313731 18413894

[B47] Li Y. F. Feng Q. Z. Gao W. Q. Zhang X. J. Huang Y. Chen Y. D. (2015). The difference between Asian and Western in the effect of LDL-C lowering therapy on coronary atherosclerotic plaque: a meta-analysis report. *BMC Cardiovasc. Disord.* 15 1–19. 10.1186/1471-2261-15-6 25971444 PMC4429819

[B48] Liao Y. Tang J. Liu T. Chen X. Hao W. (2012). Differences between smokers and non-smokers in regional gray matter volumes: a voxel-based morphometry study. *Addict. Biol.* 17 977–980. 10.1111/j.1369-1600.2010.00250.x 20731627

[B49] Lieberman M. D. Hariri A. Jarcho J. M. Eisenberger N. I. Bookheimer S. Y. (2005). An fMRI investigation of race-related amygdala activity in African-American and Caucasian-American individuals. *Nat. Neurosci.* 8 720–722. 10.1038/nn1465 15880106

[B50] Littel M. Franken I. H. A. (2007). The effects of prolonged abstinence on the processing of smoking cues: an ERP study among smokers, ex-smokers and never-smokers. *J. Psychopharmacol.* 21 873–882. 10.1177/0269881107078494 17984163

[B51] Liu P. Rigoulot S. Pell M. D. (2015). Culture modulates the brain response to human expressions of emotion: electrophysiological evidence. *Neuropsychologia* 67 1–13. 10.1016/j.neuropsychologia.2014.11.034 25477081

[B52] Luijten M. Veltman D. J. van den Brink W. Hester R. Field M. Smits M. (2011). Neurobiological substrate of smoking-related attentional bias. *Neuroimage* 54 2374–2381. 10.1016/j.neuroimage.2010.09.064 20932921

[B53] Lun J. Oishi S. Coan J. A. Akimoto S. Miao F. F. (2010). Cultural variations in motivational responses to felt misunderstanding. *Pers. Soc. Psychol. Bull.* 36 986–996. 10.1177/0146167210362979 20495092

[B54] Marianne L. Euser A. S. Munafò M. R. Franken I. H. A. (2012). Electrophysiological indices of biased cognitive processing of substance-related cues: a meta-analysis. *Neurosci. Biobehav. Rev.* 36 1803–1816. 10.1016/j.neubiorev.2012.05.001 22613258

[B55] Masuda T. Russell M. J. Chen Y. Y. Hioki K. Caplan J. B. (2014). N400 incongruity effect in an episodic memory task reveals different strategies for handling irrelevant contextual information for Japanese than European Canadians. *Cogn. Neurosci.* 5 17–25. 10.1080/17588928.2013.831819 24168198

[B56] Mcclernon F. J. Hiott F. B. Huettel S. A. Rose J. E. (2005). Abstinence-induced changes in self-report craving correlate with event-related FMRI responses to smoking cues. *Neuropsychopharmacology* 30 1940–1947. 10.1038/sj.npp.1300780 15920499 PMC1571136

[B57] Mcdonough B. E. Warren C. A. (2001). Effects of 12-h tobacco deprivation on event-related potentials elicited by visual smoking cues. *Psychopharmacology* 154 282–291. 10.1007/s002130000647 11351935

[B58] Moriguchi Y. Ohnishi T. T. Mori T. Hirakata M. Yamada M. Matsuda H. (2005). Specific brain activation in Japanese and Caucasian people to fearful faces. *Neuroreport* 16 133–136. 10.1097/00001756-200502080-00012 15671862

[B59] Moss E. M. Davidson R. J. Saron C. (1985). Cross-cultural differences in hemisphericity: EEG asymmetry discriminates between Japanese and Westerners ✰. *Neuropsychologia* 23 131–135. 10.1016/0028-3932(85)90054-5 3974852

[B60] Murata A. Moser J. S. Kitayama S. (2012). Culture shapes electrocortical responses during emotion suppression. *Soc. Cogn. Affect. Neurosci.* 49 595–601. 10.1093/scan/nss036 22422803 PMC3682443

[B61] Myrick H. Anton R. F. Li X. Henderson S. Drobes D. Voronin K. (2004). Differential brain activity in alcoholics and social drinkers to alcohol cues: relationship to craving. *Neuropsychopharmacology* 29 393–402. 10.1038/sj.npp.1300295 14679386

[B62] Nagoshi C. T. Nakata T. Sasano K. Wood M. D. (1994). Alcohol norms, expectancies, and reasons for drinking and alcohol use in a U.S. versus a Japanese college sample. *Alcohol. Clin. Exp. Res.* 18 671–678. 10.1111/j.1530-0277.1994.tb00929.x 7943674

[B63] Namkoong K. Lee E. Lee C. H. Lee B. O. An S. K. (2004). Increased P3 amplitudes induced by Alcohol-Related pictures in patients with alcohol dependence. *Alcohol. Clin. Exp. Res.* 28 1317–1323. 10.1097/01.ALC.0000139828.78099.69 15365301

[B64] Nisbett R. E. Miyamoto Y. (2005). The influence of culture: holistic versus analytic perception. *Trends Cogn. Sci.* 9 467–473. 10.1016/j.tics.2005.08.004 16129648

[B65] Nisbett R. E. Peng K. Choi I. Norenzayan A. (2001). Culture and systems of thought: holistic versus analytic cognition. *Psychol. Rev.* 108 291–310. 10.1037/0033-295X.108.2.291 11381831

[B66] Okuyemi K. S. Powell J. N. Savage C. R. Hall S. B. Nollen N. Holsen L. M. (2006). Enhanced cue-elicited brain activation in African American compared with Caucasian smokers: an fMRI study. *Addict*. *Biol.* 11 97–106. 10.1111/j.1369-1600.2006.00007.x 16759342

[B67] Patel D. R. Feucht C. (2010). Pharmacologic agents for smoking cessation: a clinical review. *Clin. Pharmacol.* 2 17–29. 10.2147/CPAA.S8788 22291484 PMC3262366

[B68] Petit G. Cimochowska A. Cevallos C. Cheron G. Kornreich C. Hanak C. (2015). Reduced processing of alcohol cues predicts abstinence in recently detoxified alcoholic patients in a three-month follow up period: an ERP study. *Behav. Brain Res.* 282 84–94. 10.1016/j.bbr.2014.12.057 25576964

[B69] Piko B. F. (2007). Motives for smoking and drinking: country and gender differences in samples of Hungarian and US high school students. *Addict. Behav.* 32 2087–2098. 10.1016/j.addbeh.2007.01.013 17317024

[B70] Ramirez J. J. Monti P. M. Colwill R. M. (2014). Alcohol-Cue exposure effects on craving and attentional bias in underage college-student drinkers. *Psychol. Addict. Behav.* 29 317–322. 10.1037/adb0000028 25243832 PMC4441600

[B71] Ray R. Schnoll R. A. Lerman C. (2009). Nicotine dependence: biology, behavior, and treatment. *Annu. Rev. Med.* 60 247–260. 10.1146/annurev.med.60.041707.160511 19630572

[B72] Robbins T. W. Ersche K. D. Everitt B. J. (2008). Drug addiction and the memory systems of the brain. *Ann. N. Y. Acad. Sci.* 1141 1–21. 10.1196/annals.1441.020 18991949

[B73] Sánchez-Johnsen L. Ahluwalia J. S. Fitzgibbon M. Spring B. J. (2006). Ethnic similarities and differences in reasons for smoking. *Addict. Behav.* 31 544–548. 10.1016/j.addbeh.2005.05.029 15982824

[B74] Schultz W. Dayan P. Montague P. R. (1997). A neural substrate of prediction and reward. *Science* 275 1593–1599. 10.1126/science.275.5306.1593 9054347

[B75] Seo D. Jia Z. Lacadie C. M. Tsou K. A. Bergquist K. Sinha R. (2011). Sex differences in neural responses to stress and alcohol context cues. *Hum. Brain Mapp.* 32 1998–2013. 10.1002/hbm.21165 21162046 PMC3236497

[B76] Shiffman S. Dunbar M. Kirchner T. Li X. Tindle H. Anderson S. (2013). Smoker reactivity to cues: effects on craving and on smoking behavior. *J. Abnorm. Psychol.* 122 264–280. 10.1037/a0028339 22708884 PMC3988583

[B77] Sinha R. Li C. S. (2007). Imaging stress- and cue-induced drug and alcohol craving: association with relapse and clinical implications. *Drug Alcohol Rev.* 26 25–31. 10.1080/09595230601036960 17364833

[B78] Suh E. Diener E. Oishi S. Triandis H. C. (1998). The shifting basis of life satisfaction judgments across cultures: emotions versus norms. *J. Pers. Soc. Psychol.* 74 482–493. 10.1037/0022-3514.74.2.482

[B79] Sul S. Choi I. Kang P. (2012). Cultural modulation of self-referential brain activity for personality traits and social identities. *Soc. Neurosci.* 7 280–291. 10.1080/17470919.2011.614001 21970690

[B80] Vecchiato G. Astolfi L. De Vico Fallani F. Toppi J. Aloise F. Bez F. (2011). On the use of EEG or MEG brain imaging tools in neuromarketing research. *Comput. Intell. Neurosci.* 2011:643489 10.1155/2011/643489 PMC3180786 21960996

[B81] Vizioli L. Foreman K. Rousselet G. A. Caldara R. (2010). Inverting faces elicits sensitivity to race on the N170 component: a cross-cultural study. *J. Vis.* 10 11–23. 10.1167/10.1.15 20143908

[B82] Vollstädt-Klein S. Hermann D. Rabinstein J. Wichert S. Klein O. Ende G. (2010a). Increased activation of the ACC during a spatial working memory task in alcohol-dependence versus heavy social drinking. *Alcohol. Clin. Exp. Res.* 34 771–776. 10.1111/j.1530-0277.2010.01149.x 20201927

[B83] Vollstädt-Klein S. Kobiella A. Buhler M. Graf C. Fehr C. Mann K. (2011a). Severity of dependence modulates smokers’ neuronal cue reactivity and cigarette craving elicited by tobacco advertisement. *Addict. Biol.* 16 166–175. 10.1111/j.1369-1600.2010.00207.x 20331560

[B84] Vollstädt-Klein S. Loeber S. Goltz C. V. D. Kirsch M. Richter A. Mann K. F. (2009). Extinction of fMRI cue-reactivity by cue-exposure based training in alcoholics. *Neuroimage* 47(Suppl. 1):S54 10.1007/s00213-015-3882-5

[B85] Vollstädt-Klein S. Loeber S. Kirsch M. Bach P. Richter A. Buhler M. (2011b). Effects of cue-exposure treatment on neural cue reactivity in alcohol dependence: a randomized trial. *Biol. Psychiatry* 69 1060–1066. 10.1016/j.biopsych.2010.12.016 21292243

[B86] Vollstädt-Klein S. Loeber S. Richter A. Kirsch M. Bach P. von der Goltz C. (2012). Validating incentive salience with functional magnetic resonance imaging: association between mesolimbic cue reactivity and attentional bias in alcohol-dependent patients. *Addict. Biol.* 17 807–816. 10.1111/j.1369-1600.2011.00352.x 21790907

[B87] Vollstädt-Klein S. Wichert S. Rabinstein J. Buhler M. Klein O. Ende G. (2010b). Initial, habitual and compulsive alcohol use is characterized by a shift of cue processing from ventral to dorsal striatum. *Addiction* 105 1741–1749. 10.1111/j.1360-0443.2010.03022.x 20670348

[B88] Wiers R. W. Boelema S. R. Nikolaou K. Gladwin T. E. (2015). On the development of implicit and control processes in relation to substance use in adolescence. *Curr. Addict. Rep.* 2 141–155. 10.1007/s40429-015-0053-z 25960940 PMC4412508

[B89] Wiers R. W. Gladwin T. E. Hofmann W. Salemink E. Ridderinkhof K. R. (2013). Cognitive bias modification and cognitive control training in addiction and related psychopathology mechanisms, clinical perspectives, and ways forward. *Clin. Psychol. Sci.* 1 192–212.

[B90] Wigmore S. W. Hinson R. E. (1991). The influence of setting on consumption in the balanced placebo design. *Br. J. Addict.* 86 205–215. 10.1111/j.1360-0443.1991.tb01770.x 2021703

[B91] Williams D. D. G. (1980). Effects of cigarette smoking on immediate memory and performance in different kinds of smoker. *Br. J. Psychol.* 71 83–90. 10.1111/j.2044-8295.1980.tb02732.x 7362935

[B92] Wray J. M. Gass J. C. Tiffany S. T. (2013). A systematic review of the relationships between craving and smoking cessation. *Nicotine Tob. Res.* 15 1167–1182. 10.1093/ntr/nts268 23291636 PMC3682845

[B93] Yarmush D. E. Manchery L. Luehring-Jones P. Erblich J. (2016). Gender and impulsivity: effects on cue-induced alcohol craving. *Alcohol. Clin. Exp. Res.* 40 1052–1057. 10.1111/acer.13030 27028602

[B94] Zane N. Sasao T. (2010). Research on drug abuse among Asian Pacific Americans. *Drugs Soc.* 6 181–209. 10.1300/J023v06n03_01

[B95] Zhang X. Chen X. Yu Y. Sun D. Ma N. He S. (2009). Masked smoking-related images modulate brain activity in smokers. *Hum. Brain Mapp.* 30 896–907. 10.1002/hbm.20552 18344177 PMC6870630

[B96] Zhang X. Salmeron B. J. Ross T. J. Geng X. Yang Y. Stein E. A. (2011a). Factors underlying prefrontal and insula structural alterations in smokers. *Neuroimage* 54 42–48. 10.1016/j.neuroimage.2010.08.008 20699124 PMC2962727

[B97] Zhang X. Salmeron B. J. Ross T. J. Gu H. Geng X. Yang Y. (2011b). Anatomical differences and network characteristics underlying smoking cue reactivity. *Neuroimage* 54 131–141. 10.1016/j.neuroimage.2010.07.063 20688176 PMC2962771

[B98] Zhu Y. Zhang L. Fan J. Han S. (2007). Neural basis of cultural influence on self-representation. *Neuroimage* 34 1310–1316. 10.1016/j.neuroimage.2006.08.047 17134915

